# Be sweet, be strong, and be tolerant: ERDL4 regulates sugar transport and promotes cold tolerance in Arabidopsis

**DOI:** 10.1093/plphys/kiad463

**Published:** 2023-08-22

**Authors:** Yee-Shan Ku

**Affiliations:** Assistant Features Editor, Plant Physiology, American Society of Plant Biologists; School of Life Sciences and Centre for Soybean Research of the State Key Laboratory of Agrobiotechnology, The Chinese University of Hong Kong, Hong Kong SAR, China

Cold stress is a major factor limiting plant growth in many parts of the world. Low temperature affects membrane structure and protein stability and reduces many enzyme activities (Ding et al. 2019). Under cold stress, plants accumulate various biological “antifreeze” compounds, including proline, anti-freezing proteins, late embryogenesis proteins, and soluble sugars (Saleem et al. 2021; Gusain et al. 2023).

Soluble sugars have diverse functions in stabilizing membranes, affecting starch-sugar conversion and eliciting sugar-signaling pathways (Satyakam et al. 2022). Cold stress also promotes the expression of several sugar transporters as part of the response for cold tolerance (Yamada and Osakabe 2017; Satyakam et al. 2022). Among other transporters, the Tonoplast Monosaccharide Transporters and Sugars Will Eventually Be Exported Transporters have been proposed to play a role in cold tolerance (Wormit et al. 2006). Nonetheless, direct evidence of sugar transport in cold tolerance is often difficult to ascertain.

In this issue, Khan et al. (2023) reported the positive role of *ERDL4* (*EARLY RESPONSE TO DEHYDRATION 6-LIKE4*) in promoting growth and cold tolerance in Arabidopsis. *ERDL4* encodes a fructose transporter located at the vacuolar membrane. The overexpression of *ERDL4* promoted cytosolic fructose accumulation. Similar to fructose treatment, the overexpression of *ERDL4* induced the expression of *TST2*, which encodes a vacuolar sugar loader. Experimental results showed that the overexpression of *ERDL4* promoted the accumulation of fructose in the cytosol as well as the accumulation of glucose in the vacuole. Using transgenic Arabidopsis, the authors showed that the overexpression of *ERDL4* promoted rosette leaf size, silique length, and seed weight (Fig. 1). Under cold stress, the overexpression also promoted the accumulation of anthocyanin in rosette leaves.

**Figure 1.**
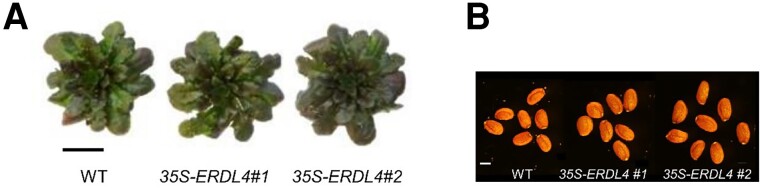
**A)** Representative images of the rosette leaves of wild type and *ERDL4* overexpressing Arabidopsis under cold stress. **B)** Representative images of the seeds of wild type and *ERDL4* overexpressing Arabidopsis under normal condition. The images were adapted and rearranged from Khan et al. 2023.

To gain more insights into the ERDL4-mediated gene regulations, through RNA sequencing the authors revealed a set of genes that were regulated by fructose while showing differential expression trends when *ERDL4* was overexpressed (Khan et al. 2023). These genes are involved in processes including starch degradation, galactose metabolism, cellulose biosynthesis, growth promotion, abscisic acid signaling, and stress responses. The results supported that the upregulation of *ERDL4* under cold stress mediates fructose transport, which signals gene regulations to achieve cold tolerance. The overall experimental results hinted at the correlation among fructose transport, anthocyanin accumulation, and cold tolerance. The study highlighted the role of fructose in cold adaptation. In addition to regulating gene expressions, the efflux of fructose to the cytosol also plays a possible role to provide precursors for anthocyanin biosynthesis.

In summary, this study by Khan et al. (2023) reported the positive role of *ERDL4* in promoting cold tolerance. ERDL4 cooperates with TST2 to promote cytosolic fructose accumulation and vacuolar glucose accumulation. ERDL4 elicits gene regulations through fructose signaling to promote plant growth and cold tolerance. In addition to reporting the role of *ERDL4* in promoting cold tolerance, the authors provide evidence to tighten the correlation among fructose transport, anthocyanin accumulation, and cold tolerance. The knowledge can be applied to crop breeding for improving the cold tolerance of crops. The cultivation of cold tolerant crops will expand the areas of land suitable for agricultural use.

## Dive Curated Terms

The following phenotypic, genotypic, and functional terms are of significance to the work described in this paper:
