# Correction to: Be sweet, be strong, and be tolerant: ERDL4 regulates sugar transport and promotes cold tolerance in Arabidopsis

**DOI:** 10.1093/plphys/kiad534

**Published:** 2023-10-13

This is a correction to: Yee-Shan Ku, Be sweet, be strong, and be tolerant: ERDL4 regulates sugar transport and promotes cold tolerance in Arabidopsis, Plant Physiology, 2023; kiad463, https://doi.org/10.1093/plphys/kiad463

In the originally published version of this article, the citation in Figure 1 legend was incorrectly given as ‘Kaur et al 2023’, instead of ‘Khan et al 2023’.

This error has been corrected.

